# A Rare Case of Duodenal Adenocarcinoma Presenting as a Subepithelial Lesion in a Patient Undergoing Investigation for Iron Deficiency Anaemia

**DOI:** 10.1155/2019/3434620

**Published:** 2019-10-07

**Authors:** Xinxin Hu, Karina Aivazian, Catriona McKenzie, May Wong, Arthur Kaffes, Payal Saxena

**Affiliations:** ^1^A.W. Morrow Gastroenterology and Liver Centre, Royal Prince Alfred Hospital, Sydney, Australia; ^2^Department of Tissue Pathology and Diagnostic Oncology, Royal Prince Alfred Hospital, Sydney, Australia; ^3^The University of Sydney, Sydney, Australia

## Abstract

**Introduction:**

Adenocarcinomas account for approximately 40% of small bowel cancers. They are typically mucosal lesions with distinctive features on endoscopy. We describe a rare case of duodenal adenocarcinoma presenting as a subepithelial lesion which posed a diagnostic challenge.

**Case:**

An 85-year-old male patient presented for investigation of iron deficiency anaemia. Initial upper endoscopy found a subepithelial duodenal lesion with central depression but otherwise normal appearing mucosa. Superficial biopsies of the duodenal lesion were unremarkable. Subsequent antegrade single balloon enteroscopy revealed active bleeding from the lesion which was refractory to endoscopic treatment. A complete local excision of the lesion via laparotomy was necessary to achieve haemostasis. Histopathology from the lesion showed a moderately differentiated duodenal adenocarcinoma with invasion into the submucosa but no evidence of lymphovascular spread.

**Conclusion:**

Duodenal adenocarcinomas are rare gastrointestinal tumours associated with a poor prognosis. This case report outlines a rare presentation of duodenal adenocarcinoma and highlights the importance of judicious assessment of lesions found on endoscopy. Advances in endoscopic diagnostic modalities could facilitate early diagnosis and improve therapeutic outcomes.

## 1. Introduction

Adenocarcinomas account for 40% of cancers in the small intestine and the most common site of involvement is the duodenum [1]. They are typically mucosal in origin and appear as polypoid or sessile lesions on endoscopy [2]. In this case report, we describe a rare presentation of duodenal adenocarcinoma as a subepithelial lesion.

## 2. Case Presentation

**Figure 1:**
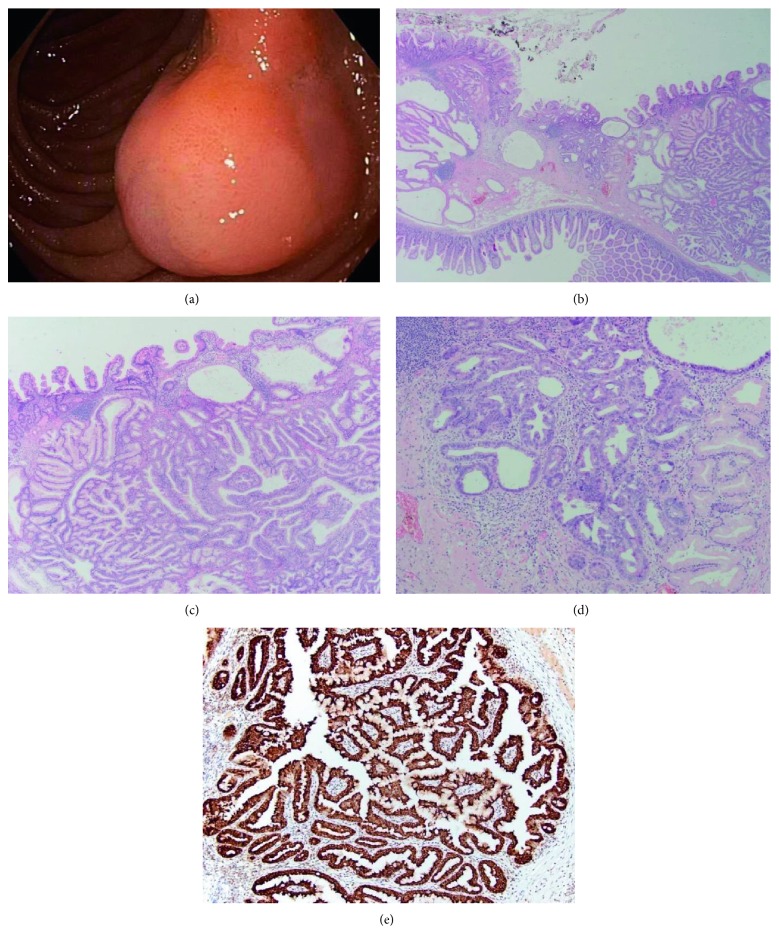
Macroscopic and microscopic views of the duodenal adenocarcinoma. (a) Appearance of the lesion on endoscopy showing central depression but otherwise unremarkable overlying mucosa. (b) *Low power*. Microphotography of the duodenal lesion with an inverted pattern of growth and normal appearing overlying mucosa. (c) *Medium power*. Microphotography of the duodenal lesion shows tightly packed tubules consistent with pyloric gland origin. (d) *Medium power*. Focus of the invasive duodenal adenocarcinoma with cytological atypia, nuclear pleomorphism, and increased nuclear to cytoplasmic ratio consistent with malignancy. (e) *Medium power*. Immunohistochemistry of the duodenal lesion shows extensive positive staining for gastric-type mucin MUC6, a marker consistent with pyloric gland origin.

An 85-year-old male patient was referred for investigation of iron deficiency anaemia. His comorbidities included ischemic heart disease and localized prostate cancer with previous transurethral resection (TURP) and external beam radiotherapy (EBRT). Gastroscopy found the presence of two Forrest grade III antral ulcers which were biopsied. Histopathology from the gastric antral biopsies showed moderate chronic gastritis and focal intestinal metaplasia. His colonoscopy was unremarkable. CT abdomen pelvis with oral and intravenous contrast did not find a source of bleeding, evidence bowel obstruction or mural thickening suspicious for malignancy. He subsequently underwent capsule endoscopy for investigation of occult gastrointestinal bleeding which found a non-bleeding lesion in the duodenum. Antegrade single balloon enteroscopy again identified the 1.5 cm polypoid, subepithelial lesion in the lateral wall of the second part of the duodenum, distal to the major papilla. The lesion had central depression and minor ulceration but otherwise normal appearing mucosa on endoscopy (Figure 1(a)). Initial biopsies of the lesion showed normal appearing intestinal mucosa with no evidence of malignancy. Subsequent enteroscopy found bleeding from the duodenal lesion, which was refractory to endoscopic therapy. Hence an open laparotomy and lateral wall excision of the lesion was performed to achieve haemostasis. Complete local excision of the lesion was successful and the entire specimen was submitted for histopathology.

The resected specimen measured 20 × 20 mm with a thickness of 6 mm. Histopathology revealed a moderately differentiated adenocarcinoma with submucosal invasion, arising in an adenomatous polyp with an inverted pattern of growth and normal appearing mucosa (AJCC 8^th^ edition, Stage I, T1bN0M0) (Figures 1(b) and 1(c)). The focus of the duodenal adenocarcinoma featured prominent cytological atypia, nuclear pleomorphism and increased nuclear to cytoplasmic ratio consistent with malignancy (Figure 1(d)). Immunohistochemistry of the lesion showed positive staining for gastric-type mucins MUC5AC and MUC6 (Figure 1(e)). The lesion was negative for MUC1, MUC2, CK20, and had patchy staining for CDX2 and CK7. Staining for DNA mismatch-repair proteins (MSH2, MLH1, MSH6, and PMS2) showed normal levels of expression. The histological features and immunohistochemistry profile of the lesion is most consistent with a tumour of pyloric gland origin.

## 3. Discussion

Duodenal adenocarcinomas are rare but aggressive tumours that carry a poor prognosis [3]. They typically arise from the mucosal layer and appear as polypoid, sessile, stenotic, ulcerative, or infiltrative lesions on endoscopy [2]. This case report describes a rare presentation of duodenal adenocarcinoma as a subepithelial lesion, which evaded diagnosis on initial superficial biopsies.

Endoscopy is the initial diagnostic modality of choice as it allows direct visualization and biopsy of the lesion for tissue diagnosis. A number of different biopsy techniques are available depending on the layer of the gastrointestinal tract involved. As demonstrated in our case, conventional biopsy forceps have a low diagnostic yield for subepithelial lesions [4]. Partial resection and unroofing techniques improve the diagnostic yield, but are linked to increased rates of haemorrhage, perforation, and perilesion fibrosis [5]. These techniques would have been difficult to implement in our patient as the lesion was located in the distal duodenum and required balloon assisted enteroscopy to access.

Endoscopic ultrasound (EUS) has demonstrated utility in the assessment of subepithelial lesions found on endoscopy. It provides a minimally invasive method of acquiring tumour characteristics such as size, layer of infiltration, and the presence of local lymphadenopathy which holds prognostic value and guides the use of biopsy techniques. EUS itself can be combined with fine needle aspiration (EUS-FNA) and fine needle biopsy (EUS-FNB) techniques for the sampling of lesions arising from the submucosa and muscularis propria [6]. EUS-FNB has been shown to be equally as safe as EUS-FNA with the added advantages of larger tissue acquisition and preservation of cellular architecture, which aids in histopathological diagnosis [7]. However, the distal location of our lesion would hinder access with an echoendoscope for EUS and biopsy.

The treatment approach for duodenal adenocarcinoma differs from that of other subepithelial lesions in the small bowel. The American Society of Gastrointestinal Endoscopy (ASGE) outlines an approach for the management of subepithelial lesions, which takes into account histological features, size, layers involved, distal spread, symptomatology, and other patient factors [5]. Endoscopic management is generally favoured for tumours less than 2 cm in size, whereas surgery is favoured for tumours greater than 4 cm. Management for lesions between 2 and 4 cm is dependent on additional factors including appearance on EUS [5]. In contrast, duodenal adenocarcinomas are aggressive tumours and surgical resection remains the current standard of care. Surgical approaches range from local excision to pancreaticoduodenectomy depending on tumour site and other patient factors [8]. Assessment of lymphatic spread and lymphadenectomy should be considered as nodal metastasis has been shown to be a negative prognostic factor in duodenal adenocarcinoma [9]. The contrasting approaches to management of duodenal adenocarcinoma as compared to other subepithelial lesions highlight the importance of accurate tissue diagnosis in improving patient outcomes.

Although rare, there have been previous reports of duodenal adenocarcinoma presenting as a subepithelial lesion. Kojima et al. reported this presentation in a 63-year-old female patient undergoing investigation for epigastric pain [10]. The lesion was found in the second part of the duodenum with an ulcerated surface but otherwise normal appearing mucosa. There was strong clinical suspicion of malignancy prior to endoscopy as the lesion was palpable on physical examination and there were concerning radiological and biochemical features. However, initial endoscopic biopsies also failed to adequately diagnose the lesion, revealing only inflammatory changes in the mucosa. The histopathological diagnosis was only made following excision of the lesion as in our case. Together, these two cases describe a rare presentation of duodenal adenocarcinoma and highlights the importance of vigilance when assessing lesions found on endoscopy.

## 4. Conclusion

This case report describes an unusual presentation of duodenal adenocarcinoma as a subepithelial lesion with initially unremarkable superficial biopsies. The diagnosis was only made retrospectively following complete excision of the lesion. The failure of initial biopsies to successfully diagnose the underlying lesion exposes the limitations of current techniques in the sampling of subepithelial lesions not accessible via endoscopic ultrasound. Improvements in endoscopic modalities for the assessment of subepithelial lesions could facilitate early diagnosis and improve therapeutic outcomes.
